# Surgical Asepsis

**Published:** 1903-09

**Authors:** 


Surgical Asepsis.—Especially Adapted to Operations in the Home of the Patient. By Henry B. Palmer, M. D., Consulting Surgeon in the Central Maine General Hospital. 90 illustrations, 231 pages, large 12mo, extra cloth. Price, $1.25 net, delivered. F. A. Davis Company, Publishers, 1914-16 Cherry Street, Philadelphia.

One of the greatest drawbacks to the practice of surgery is the dread on the part of many patients to leave home and go to a hospital, especially if the operation be a serious one. The fear of dying away from home and friends is liable to work upon the mind of the patient so as to prevent his giving his consent to a needed operation. Moreover, should he consent to being taken to a hospital, convalescence may be hindered by a constant desire to go home, or worse still by a too early removal thence. Unquestionably, then, home operations are to be preferred, provided the necessary hygienic and aseptic conditions can be obtained.

In this ingenious little book the author endeavors to show that home surgery has a much wider application than it is generally conceded. With the assistance of trained nursing and proper instructions, the physician can convert some one room in the most of our homes into a veritable little operating room.

The book covers the ground well, is short, concise, and above all, practical, and should be in the hands of every physician who practices surgery or who frequently has the after care of surgical cases.

